# The cost effectiveness of an early transition from hospital to nursing home for stroke patients: design of a comparative study

**DOI:** 10.1186/1471-2458-10-279

**Published:** 2010-05-26

**Authors:** Ron WH Heijnen, Silvia MAA Evers, Trudy DEM van der Weijden, Martien Limburg, Jos MGA Schols

**Affiliations:** 1 Nursing Home Physician, Vivre Group, Polvertorenstraat 6, 6211 LX Maastricht, The Netherlands; 2 School of CAPHRI/Department of Health Organisation Policy and Economics, Maastricht University, Maastricht, The Netherlands; 3 School of CAPHRI/Department of General Practice, Maastricht University, Maastricht, The Netherlands; 4 Department of Neurology, Maastricht University Medical Centre, Maastricht, The Netherlands; 5 School of CAPHRI/Department of General Practice/Nursing Home Medicine, Maastricht University, Maastricht, The Netherlands

## Abstract

**Background:**

As the incidence of stroke has increased, its impact on society has increased accordingly, while it continues to have a major impact on the individual. New strategies to further improve the quality, efficiency and logistics of stroke services are necessary. Early discharge from hospital to a nursing home with an adequate rehabilitation programme could help to optimise integrated care for stroke patients.

The objective is to describe the design of a non-randomised comparative study evaluating early admission to a nursing home, with multidisciplinary assessment, for stroke patients. The study is comprised of an effect evaluation, an economic evaluation and a process evaluation.

**Methods/design:**

The design involves a non-randomised comparative trial for two groups. Participants are followed for 6 months from the time of stroke. The intervention consists of a redesigned care pathway for stroke patients. In this care pathway, patients are discharged from hospital to a nursing home within 5 days, in comparison with 12 days in the usual situation. In the nursing home a structured assessment takes place, aimed at planning adequate rehabilitation. People in the control group receive the usual care. The main outcome measures of the effect evaluation are quality of life and daily functioning. In addition, an economic evaluation will be performed from a societal perspective. A process evaluation will be carried out to evaluate the feasibility of the intervention as well as the experiences and opinions of patients and professionals.

**Discussion:**

The results of this study will provide information about the cost effectiveness of the intervention and its effects on clinical outcomes and quality of life. Relevant strengths and weaknesses of the study are addressed in this article.

**Trial registration:**

Current Controlled Trails ISRCTN58135104

## Background

By the year 2020, 250 per 100,000 patients in the Netherlands will suffer from a stroke, and in many instances this will result in permanent disabilities and handicaps[1]. Substantial evidence is available showing that hospital stroke units reduce mortality, dependence and institutionalisation[2] and that better outcomes are associated with comprehensive and early assessment of stroke patients[3]. It is also suggested that organised integrated stroke care and the use of early supported discharge services for stroke patients are less expensive than general medical care, due to a reduction in hospital stay[4,5]. However there are very few data on the cost effectiveness of integrated stroke care from a societal point of view[6].

In the Netherlands 32% of stroke patients return directly to their home after hospital stay, 9% are discharged to a rehabilitation centre and 31%, mainly elderly stroke patients, are discharged to a nursing home for rehabilitation[7]. Consequently, contrary to nursing homes in many other countries, Dutch nursing homes fulfil an important role in the rehabilitation of stroke patients[8]. The mean duration of hospital stay of stroke patients in 2004 was 12.6 days[9]. Demographic developments, the increased incidence and prevalence of stroke, the emergence of disease management programs, and changes in the structure of the Dutch health care system have led to new strategies to further improve the quality, efficiency and logistics of care processes. Optimizing stroke services involves: a) faster admission of stroke patients to the hospital, leading to improved chances for effective intervention, b) early discharge from hospital, with an adequate plan for rehabilitation and c) improving care after discharge to home.

In 2006, these developments led to a redesign of the Stroke Service Maastricht Heuvelland. The essentials of this redesign are: stroke patients are admitted to Maastricht University Medical Centre for a maximum of 5 days for diagnosis, early intervention and stabilization, after which they are discharged to a special assessment and rehabilitation ward in a nursing home. In this nursing home, stroke patients undergo a structured multidisciplinary assessment, lasting a maximum of 5 days, and take part in their first rehabilitation activities. During assessment, the appropriate follow-up treatment is determined. Patients are then admitted to the follow-up setting for rehabilitative care. This means discharge to either their own home situation, to a specific rehabilitation hospital, or continued stay in the nursing home for prolonged rehabilitation or continuing stay.

Cost effective integrated stroke care requires a high degree of coordination between professionals in hospitals, nursing homes and home care, a high quality integral assessment in the nursing home and a system of adequately timed patient transitions.

The principal expectation is that the redesigned process of the Stroke Service Maastricht Heuvelland will lead to cost effective care, with expected improvement in the quality of care as well.

This article describes the design of a longitudinal comparative study, evaluating the cost effectiveness of an early discharge to and assessment of stroke patients in a nursing home, as part of a redesigned integrated stroke care programme, in comparison with a comparable stroke service region, where the redesign has not been implemented.

The research questions in this study are:

1. What is the effect of early admission to and assessment in the nursing home on functional outcomes, quality of life, and satisfaction with care in comparison with the usual care provided by a stroke service?[Effect evaluation]

2. From a societal perspective, what is the incremental cost effectiveness of early admission to and assessment in the nursing home, in comparison with the usual care provided by a stroke service?[Economic evaluation]

3. Research questions for the process evaluation

a) Is the new care pathway executed on time according to the protocol?[Process evaluation]

b) What are the experiences and opinions of patients and professionals about the newly developed care pathway?[Process evaluation]

## Methods/Design

### Study Design

We will conduct a comparative study with retrospective and prospective parts, in which we will compare the redesigned stroke service in the intervention region with a comparable region that offers the usual stroke care.

### Ethical approval and informed consent

The Medical Ethical Committee of the University of Maastricht has granted ethical approval. The trial is registered as ISRCTN58135104.

An information brochure will be given to all eligible patients. At inclusion, patients will be informed personally and also by means of written information on all aspects of the project. The privacy of the participating patients is protected, and all data will be coded and processed anonymously. It will be made clear in the informed consent form that each patient can terminate his or her participation in the trial at any moment without the care being influenced.

### Study population

The patient population consists of consecutive stroke patients who are admitted to the hospitals in both research regions during a period of 18 months. The diagnosis of stroke will be made by a neurologist based on patient history, physical examination and neuro-imaging. Patients will be eligible to participate if they meet the following criteria: over 18 years of age and fluent in Dutch. Exclusion criteria are: a life expectancy of less then a few days, a previous diagnosis of dementia, hospital discharge to home within a few days and occurrence of complications which require prolonged hospital care. Patients suffering a recurrent stroke during their participation in this study will not be asked to participate a second time.

### Sample Size

The primary goal of this study is to achieve cost reduction without loss of quality of life. Based on an earlier study among stroke patients[10], we estimate a difference in utilities indicating health related quality of life between the 2 regions. In this earlier study by Olsen, the utility difference based on the EuroQol was 0.11. Based on a power of 80%, alpha 0.05, our study will need a sample size of 111 participants per group.

We expect a 25% drop-out of patients between inclusion and the follow-up meeting 26 weeks later, due to premature termination of the trial participation, inability to cooperate in the trial, or mortality[11]. To correct for this expected drop-out, the number of patients included will be increased to 139 participants per group.

### Intervention

**Figure 1:**
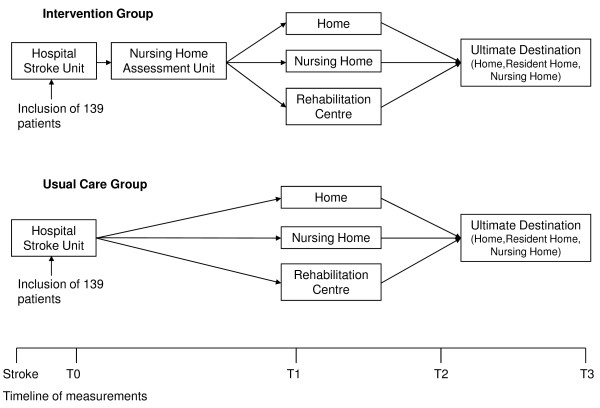
**Flowchart of the study**.

The intervention (figure 1) consists of the execution of a redesigned care pathway for stroke patients admitted to the Maastricht University Medical Centre. Every patient with a suspected stroke will be analysed in the emergency ward. In case of a stroke, the patient will be admitted to the stroke unit of the hospital, where, if indicated, thrombolysis will be followed by further diagnosis and treatment. The new aspect of the critical care pathway consists of a strict discharge regime in the neurology department of the hospital. All necessary testing and treatment in the hospital can be performed within 5 admission days, after which patients may be discharged if medically stable. The underlying assumption for the design is that hospitals are specialized in acute care and treatment and do not provide optimal rehabilitation facilities. From a cost effectiveness point of view as well, it seems more appropriate to provide rehabilitation in a centre specialised for this purpose.

In the redesigned care pathway, after 5 days all stroke patients are discharged to a nursing home with a specialised assessment unit, resulting in a tailored rehabilitation programme. Only patients who can be discharged directly to their home within five days, or patients with complications requiring prolonged hospital care, will not be referred to the specialised nursing home unit. The nursing home physician examines each patient immediately on arrival in the nursing home and initiates the assessment program. In this program a multidisciplinary team, consisting of a psychologist, physiotherapist, occupational therapist, speech therapist, and trained nurses, examine the patient by means of a structured assessment protocol. Following assessment, the team will meet within five days of the patient's admission to make recommendations for the best rehabilitation program. Their advice will be based on admission and discharge criteria formulated by the various care providers participating in the stroke service. A structured possibility for the nursing home physician to consult a rehabilitation physician, if needed, is arranged. After the multidisciplinary meeting the patient and his family will be informed about the proposed rehabilitation track and after their approval this track can be started.

There are three options for rehabilitation after the assessment.

1. Rehabilitation at home with outpatient care provided by therapists from primary healthcare or day care rehabilitation in a hospital or nursing home

2. Inpatient rehabilitation in a nursing home

3. Inpatient rehabilitation in a rehabilitation centre

### Usual care

The redesigned stroke service provided by the Stroke Service Maastricht Heuvelland, the intervention region, will be compared to "care as usual" provided by the stroke service in the Eindhoven area.

In the Stroke Service Eindhoven stroke patients are admitted to the stroke unit of the Catharina hospital in Eindhoven, where diagnostic tests, treatment and observation take place. During the patients' stay in the hospital, an assessment is performed in order to determine the best suitable rehabilitation facility for the stable patient. A physiotherapist, an occupational therapist and trained nurses carry out the assessment; if necessary they are supported by a speech therapist or a psychologist.

On the basis of admission and discharge criteria formulated by the various care providers who participate in the Stroke Service Eindhoven, the patient can be discharged to home, to a rehabilitation centre or to one of four nursing homes participating in the stroke service. The mean duration of the hospital stay in Eindhoven is 10 days. Consequently, the main differences in care arrangements between the experiment and the control region are the early hospital discharge and the structured assessment in the nursing home.

### Effect evaluation

The primary outcome measures of the effect evaluation are quality of life and activities of daily life (ADL). Quality of life is measured by means of the Stroke Adapted Sickness Impact Profile 30 (SA-SIP 30)[12] and ADL by means of the Barthel index (BI)[13,14].

Secondary outcome measures are: instrumental activities of daily living (IADL) measured by means of the Frenchay Activity Index (FAI)[15], handicap measured by means of the Modified Rankin Scale (MRS)[16]. Cognitive functioning is measured by means of Mini Mental State Examination (MMSE)[17], Apraxia Test (AT)[18] and Star Cancellation Test (SCT)[19]. Anxiety and depression are measured by the Hospital Anxiety and Depression Scale (HADS)[20] and the patients' satisfaction with stroke care is measured by means of the Satisfaction with Stroke Care Questionnaire (SASQ-19)[21]. The strain on caregivers is measured by the Caregivers Strain Index (CSI)[22].

Other secondary outcome measures are medical complications occurring within 3 months after stroke. The following diagnoses are regarded as medical complications: a new stroke, epileptic seizures, pneumonia, urinary tract infections, fractures, bedsores, myocardial infarct, heart failure and atrial fibrillation. The data on medical complications will be collected from the patients' files.

Besides the primary and secondary outcome measures we assess some background variables which are considered to be predictors, confounders or effect modifiers. The following personal characteristics are assessed: age, sex, socio-economic status, risk factors, co-morbidity, stroke location and stroke severity measured by the National Institute of Health Stroke Scale (NIHSS)[23]. All background variables are measured at baseline.

### Economic evaluation

The economic evaluation compares costs and outcomes of stroke care given in the Stroke Service Maastricht Heuvelland to care as usual provided by the Stroke Service Eindhoven. The economic evaluation will involve a combination of a cost-effectiveness analysis (CEA) and a cost-utility analysis (CUA). In a CEA effects are presented in clinical outcomes.

The primary outcome measure of the cost-effectiveness analysis will be the SASIP-30. Within the cost-utility analysis, outcomes will be measured by means of the standard Dutch version of the EuroQol (EQ-5 D)[24]. This is a self-administered questionnaire, which will be completed together with the cost questionnaire at 12 weeks and 26 weeks.

Both generic quality of life, as well as utilities, will be derived by means of the EQ-5 D. The EQ-5D is chosen because it is a widely used quality of life instrument. The EQ-5D contains 5 dimensions of health-related quality of life; mobility, self-care, daily activities, pain/discomfort and anxiety/depression. Each can be rated at three levels: no problems, some problems and major problems. The 5 dimensions can be added to comprise an overall health state. This health state will be translated into a number, a utility. Utility values can be calculated for these health states, using preferences elicited from a general population, the so-called Dolan algorithm[25]. The utility values derived from the Dolan algorithm will be used to compute Quality Adjusted Life Years (QALYs). The Dolan algorithm has been established using a general population from the UK. Recently a Dutch algorithm has become available, and this will be used in the sensitivity analysis[26]. The utilities at the two time points are used to compute a Quality Adjusted Life Years (QALY) score by means of the area under the curve method. Furthermore, the EQ-5D consists of a visual analogue scale (VAS) ranging from zero (worst imaginable health state) to 100 (best imaginable health state). The reliability and validity of the EQ-5D has been established[27]. The primary outcome measure for the cost-utility analysis will be Quality Adjusted Life Years (QALYs), based on the EuroQol utility scores[24,27]. In the CUA, the incremental cost-effectiveness ratio (ICER) will be expressed as the incremental costs per QALY. This economic evaluation will be performed from a societal perspective, which implies that all relevant costs and outcomes will be taken into account.

### Process evaluation

The process evaluation consists of two parts. The first part of the process evaluation consists of assessing whether care after the redesign was performed in the time planned. For this part of the process evaluation, the patients' files will be screened for protocol violations related to the discharge time from hospital, according to protocol, and the duration of assessment in the nursing home. If possible the reasons for these protocol violations will be collected from the patients' files as well.

The second part assesses how patients, their personal caregivers and professionals experience care after the redesign. Data will be collected from 20 patients and from their personal caregivers. Data will also be collected from 20 professionals working in the redesigned care system. The data will be collected by means of semi-structured in-depth interviews. The patients and professionals will be selected by means of purposeful sampling, to ensure that the interviewees are heterogeneous on relevant determinants such as age, severity of disease, level of functioning, housing situation (living alone, stairs, etc.). The patients will be interviewed at home or in the institution were they are being cared for.

To ensure the open character of the interviews they will be held by a person who is not related to the direct project group. All interviews will be audiotaped and transcribed verbatim.

### Instruments

The instruments used in this study are shown in Table 1

**Table 1:** Overview of instruments per time point

	Time after stroke			
Instrument	T0	T1	T2	T3
	< 1 week	4 Weeks	12 Weeks	26 Weeks
SA-SIP30			X	X
Barthel index	X	X	X	X
Frenchay Activities Index	X			X
Modified Rankin Scale	X	X	X	X
Mini Mental State Examination	X			X
Apraxia Test	X			X
Star Cancellation Test	X			X
Hospital Anxiety and Depression scale		X		X
SASC-19	After every discharge			
Caregivers Strain Index				X
EQ-5D			X	X
Cost Questionnaire			X	X

Instruments used for the screening process, the clinical outcome assessment and the economic evaluation. In Table 1 an overview of all assessments per time point is shown.

#### Stroke Adapted Sickness Impact Profile 30 (SA-SIP30)

The Stroke Adapted Sickness Impact Profile measures sickness specific quality of life in stroke patients and is a modified version of the 136-item SIP. This instrument was developed primarily to overcome the length of the original SIP, which is its major disadvantage. The SA-SIP30 is a 30-question instrument with eight subgroups, created by eliminating the most irrelevant questions from the initial test. The higher the score the lesser the quality of life after stroke[12].

#### Barthel index (BI)

The Barthel index is a generic questionnaire which consists of 10 items measuring activities of daily life (ADL) and mobility. A high score on the Barthel index corresponds with a high degree of independence concerning the activities of daily life[13,14].

#### Frenchay Activities Index (FAI)

The Frenchay Activities Index is a stroke specific instrument to assess functional status. The FAI is comprised of 15 items, each concerning an activity that requires some decision making and organisation on the part of the patient[15].

#### Modified Rankin Scale (MRS)

The Modified Rankin Scale is a widely used instrument that measures levels of handicap. It defines 6 levels of disability, ranging from 0 (no symptom) to 5 (severe disability, bedridden, incontinent and receiving constant nursing care and attention)[16].

#### Mini Mental State Examination (MMSE)

The mini mental state examination is the most widely used instrument to screen for cognitive dysfunction. The MMSE assesses orientation, memory, attention, language, and constructive functions. The MMSE consists of 20 items with a maximal total score of 30[17].

#### Apraxia Test (AT)

The Apraxia Test is a short and easy test to measure the degree of apraxia in stroke patients. It consists of two subtests, one designed to evaluate the use of objects and another to evaluate the ability to imitate gestures. The maximum score for the total test is 90[18].

#### Star Cancellation Test (SCT)

The Star Cancellation Test is the most sensitive single test for neglect. Depending on the number of missed stars the magnitude of neglect can be measured[19].

#### Hospital Anxiety and Depression Scale (HADS)

The Hospital Anxiety and Depression scale was developed to identify anxiety and depression among patients. It is divided into an anxiety subscale and a depression subscale, each containing seven intermingled items. The maximum score is 47. The higher the score the greater the possibility of anxiety or depression[20].

#### Satisfaction with Stroke Care Questionnaire 19 (SSCQ-19)

The 19-item version of the satisfaction with stroke care questionnaire is comprised of eight items measuring satisfaction with inpatient stroke care, and eleven items measuring satisfaction with stroke care after discharge[21].

#### Caregivers Strain Index (CSI)

The Caregivers Strain Index is a 13-item instrument that ascertains strain on care givers across domains of employment, finances, physical health, and social relationships. A positive answer to 7 or more of the items reflects a more than average strain on caregivers[22].

#### The National Institute of Health Stroke Scale (NIHSS)

The National Institute of Health Stroke Scale provides a measure of severity of symptoms associated with cerebral strokes. It measures level of consciousness, visual fields, motor response, sensation, language and neglect on weighted scales. The NIHSS can be used with persons of all ages, including geriatric patients, who have had a stroke[23]. The higher the score, the more severely affected is the patient.

#### European Quality of Life instrument (EuroQol)

The European Quality of life instrument (EuroQol) is a well-known generic instrument measuring health-related quality of life. It includes 5 dimensions (mobility, self-care, daily activities, pain/discomfort and anxiety/depression) and a visual analogue scale that evaluates patients' perceived health status[24]. Each dimension can be rated at three levels: no problems, some problems and major problems. The 5 dimensions can be summarized in a health state.

#### Cost Questionnaire

We will assess intervention costs, healthcare costs, patient and family costs, and costs outside the healthcare sector. For this study we will develop a cost questionnaire especially designed for this group, based on existing questionnaires[28,29], which identify all relevant costs aspects.

### Effect analysis

For the analyses we will use SPSS statistical software and Excel (for the Bootstraps). Missing data on the item level will be handled using SPSS missing value analysis. If considerable data are missing related to specific instruments, imputation will be considered.

A baseline analysis will be performed to examine the comparability of groups at baseline for both costs and outcomes. If necessary, methods will be applied to correct for differences in baseline[30]. A Kolmogorov-Smirnov test will be performed to investigate whether the data are normally distributed. If the data are distributed normally, our primary analysis will start with a t-test. If data are skewed, the primary analysis will be based on a non-parametric test for assessing two independent samples, i.e. a Mann-Whitney U test.

As it is known that in non-randomised comparative studies, variations in case mix between centres can influence the interpretation of outcome data[31], we would like to explore this in further analysis. Therefore, for each of the data sets collected at all measurement points, differences in outcome variable between the 2 regions will be tested using multiple MANCOVAs, entering various indicators of case mix as co-variates, i.e. age, gender, stroke severity. In addition, information on possible confounding factors and effect modifiers will be collected and analysed.

### Economic analysis

Total costs will be estimated using a bottom-up (or micro-costing) approach, where information on each element of service used is multiplied by an appropriate unit cost and these are added to provide an overall total cost[32]. We will assess intervention costs, healthcare costs, patient and family costs, and costs outside the health care sector. For this study we will use a cost questionnaire especially designed for this group, based on existing questionnaires[28,29], which will identify all relevant cost aspects.

To measure the actual use of resources, data will be obtained using combined sources (registrations by professionals and cost questionnaire). Resources used relating to the interventions will be an estimation of the time spent by the professionals, based on prospective registration in a random sample. All use of resources by the patient and their family, in and outside the health care sector, will be measured by means of a cost questionnaire, in which the resource utilization is recorded at 12 weeks and 26 weeks during the follow-up period. These sources of information will be combined.

The valuation of healthcare costs and costs to patient and family will be based on the updated Dutch manual for cost analysis in healthcare research[33,34]. This manual recommends using standardized cost prices. In brief, the manual recommends that prices of informal care will be based on shadow prices for unpaid work (meaning a standard cost price based on general hourly wages). Costs of transport will be calculated as the mean distance per destination multiplied by standard cost prices. Costs of medication will be calculated using prices based on Daily Defined Dosage (DDD) taken from the Dutch Pharmacotherapeutic Compass[35], indicating the mean medication usage per adult per day. Productivity costs will be calculated by means of the friction costs method, based on a mean added value of the Dutch working population. The friction costs method takes into account production losses confined to the period needed (usually 90 days) to replace a sick employee. In case of uncertainty we will use a conservative estimation (i.e. the lowest cost price). Cost prices will be expressed in 2010 euros. If necessary, existing cost prices will be updated to 2010 using the consumer price index (CPI)[33,34].

Despite the usual skewness in the distribution of costs, arithmetic means are generally considered the most appropriate measure to describe cost data[36,37]. Therefore, arithmetic means (and standard deviations) will be presented. In case cost data are skewed, non-parametric bootstrapping will be used to test for statistical differences in costs between the intervention and control group. Non-parametric bootstrapping is a method based on random sampling with replacement based on the individual data of the participants[38]. The bootstrap replication will be used to calculate 95% confidence intervals around the costs (95% CI), based on the 2.5 th and 97.5 th percentiles. If cost data are distributed normally, t-tests will be used.

The incremental cost effectiveness ratio (ICER) will be determined on the basis of incremental costs and effects of the intervention compared to care as usual. The cost effectiveness ratio will be stated in terms of costs per outcome rate; the cost utility ratio will focus on the net cost per QALY gained.

The ICER will be calculated as follows. ICER = (Ci-Cc)/(Ei-Ec), where Ci represents the total costs of the intervention group at the 26-weeks follow-up, Cc the total costs of the care as usual group at the 26-weeks follow-up, Ei the effects at the 6-month follow-up for the intervention group and Ec the effect at the 26-weeks follow-up for the care as usual group. The robustness of the ICER will be checked by non-parametric bootstrapping (1000 times). Bootstrap simulations will also be conducted in order to quantify the uncertainty around the ICER, yielding information about the joint distribution of cost and effect differences. The bootstrapped cost-effectiveness ratio will be plotted subsequently in a cost-effectiveness plane, in which the vertical line reflects the difference in costs and the horizontal line reflects the difference in effectiveness.

The choice of treatment depends on the maximum amount of money that society is prepared to pay for a gain in effectiveness, which is called the ceiling ratio. Therefore, the bootstrapped ICERs will also be depicted in a cost effectiveness acceptability curve showing the probability that the intervention care is cost effective using a range of ceiling ratios.

### Process evaluation analysis

The process evaluation will be analysed mainly by means of qualitative data analysis. The interviews will be analysed by directed content analysis[39]. After identifying and coding text passages relevant to the research question, the descriptive codes will be compared and contrasted by sequential and retrospective searching within and among the interviews. The codes will be grouped into larger themes, explored further, structured, refined and reduced in number. Data will be collected and analysed concurrently, allowing both expected and emergent themes and ideas to be incorporated and explored in subsequent interviews. Units of text referring to similar codes will be grouped and categorized systematically by one central coder, who is coding all the interviews. For the richest interviews - in the opinion of the interviewer - a full open coding of the transcript will be independently executed by the central coder and the interviewer. Differences in coding will be resolved by consensus discussion face-to-face or by phone. The central coder will then analyse the other interviews in the subset of interviews done by the one interviewer, and the interviewers will check the coding.

## Discussion

Implementation of the redesigned stroke care pathway in the Stroke Service Maastricht Heuvelland started in 2006. It has yet to be seen whether the introduction of this care pathway has led to improvement of the quality of care for stroke patients. The results of this study will provide information about the cost effectiveness of the intervention and its effects on clinical outcomes and quality of life. In this respect the relevance of this study lies in the fact that it is one of the first studies assessing the cost effectiveness of a stroke service from a societal point of view. In case of proven cost effectiveness, arguments for implementing the intervention into usual healthcare are clear and evident.

A weak point of this study is the possible bias of non-randomisation. In our study randomisation is impossible as the location of the stroke patient necessarily determines to which hospital the patient will be admitted, and in order to prevent contamination effects, only one treatment will be offered in one hospital. If our intervention appears to be cost effective, the next step will be broader implementation in more nursing homes. In addition, it will be possible to perform a cluster randomised trial to obtain even more evidence on the effectiveness of the interventions. A strong point of the study is the standardised and consequent use of validated measurement instruments, which make the characteristics of the study population accessible for further studies.

## Competing interests

The authors declare that they have no competing interests.

## Authors' contributions

All authors participated in the design of the study. RWHH drafted the manuscript, is involved in developing the intervention and selecting the measurement instruments, as well as in the implementation, analysis and reporting aspects of the study. SMAAE is involved in all aspects of the study and advises the project team with regard to the economic evaluation. TDEMvdW is involved in all aspects of the effect en process evaluation. ML is involved in developing the intervention and advised with regard to measurement instruments. JMGA is involved in all aspects of the study, especially the design of the study and in the development of the intervention, as well as in the drafting of the manuscript. All authors contributed to the writing of the manuscript and approved the final version of the manuscript.

## Pre-publication history

The pre-publication history for this paper can be accessed here:

http://www.biomedcentral.com/1471-2458/10/279/prepub
